# Alpine grassland plants grow earlier and faster but biomass remains unchanged over 35 years of climate change

**DOI:** 10.1111/ele.13474

**Published:** 2020-02-12

**Authors:** Hao Wang, Huiying Liu, Guangmin Cao, Zhiyuan Ma, Yikang Li, Fawei Zhang, Xia Zhao, Xinquan Zhao, Lin Jiang, Nathan J. Sanders, Aimée T. Classen, Jin‐Sheng He

**Affiliations:** ^1^ State Key Laboratory of Grassland Agro‐Ecosystems College of Pastoral Agriculture Science and Technology Institute of Innovation Ecology Lanzhou University Lanzhou 730000 China; ^2^ Institute of Ecology College of Urban and Environmental Sciences Key Laboratory for Earth Surface Processes of the Ministry of Education Peking University Beijing 100871 China; ^3^ Key Laboratory of Adaptation and Evolution of Plateau Biota Northwest Institute of Plateau Biology Chinese Academy of Sciences Xining 810008 China; ^4^ State Key Laboratory of Vegetation and Environmental Change Institute of Botany Chinese Academy of Sciences Beijing 100093 China; ^5^ School of Biological Sciences Georgia Institute of Technology Atlanta Georgia 30332 USA; ^6^ Environmental Program Rubenstein School of Environment and Natural Resources University of Vermont Burlington VT 05405 USA; ^7^ Rubenstein School of Environment and Natural Resources University of Vermont Burlington VT 05405 USA; ^8^ Gund Institute for Environment University of Vermont Burlington VT 05405 USA

**Keywords:** alpine grassland, biomass production, climate warming, ecosystem function, functional group composition, phenology, plant growth, the Tibetan Plateau

## Abstract

Satellite data indicate significant advancement in alpine spring phenology over decades of climate warming, but corresponding field evidence is scarce. It is also unknown whether this advancement results from an earlier shift of phenological events, or enhancement of plant growth under unchanged phenological pattern. By analyzing a 35‐year dataset of seasonal biomass dynamics of a Tibetan alpine grassland, we show that climate change promoted both earlier phenology and faster growth, without changing annual biomass production. Biomass production increased in spring due to a warming‐induced earlier onset of plant growth, but decreased in autumn due mainly to increased water stress. Plants grew faster but the fast‐growing period shortened during the mid‐growing season. These findings provide the first *in situ* evidence of long‐term changes in growth patterns in alpine grassland plant communities, and suggest that earlier phenology and faster growth will jointly contribute to plant growth in a warming climate.

## Graphical abstract

Climate change reshapes plant growth patterns by shifting phenology earlier, enhancing growth rate, and shortening growth period in a Tibetan alpine grassland over 35 years. The changes in growth patterns alters seasonal, but not annual, biomass production.

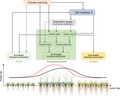

## Introduction

Plants are known to respond to the seasonality of environmental conditions such as temperature, precipitation, radiation and day length (Cleland *et al.* 2007; Piao *et al.* 2019). At mid to high latitudes, plant growth usually increases at the beginning of the growing season in spring, reaches its maximum during the mid‐growing season, and declines towards the end of the growing season in autumn, a pattern that is often temporally compressed at higher latitudes and altitudes (Billings & Mooney 1968; Körner 2003). Plant growth patterns (i.e., phenology and growth rate) respond to climate variation (Jonas *et al.* 2008; Wingler & Hennessy 2016) and have an important role in regulating Earth’s climate because they drive seasonal land‐atmosphere exchange of carbon, water and energy (Wang *et al.* 2011; Buitenwerf *et al.* 2015; Xia *et al.* 2015). Plant growth patterns also influence the ability of plant communities to provision animals with habitats and food resources (Hegland *et al.* 2009; Gonsamo *et al.* 2018).

Recent climate warming is generally expected to alleviate low temperature constraints on plant growth in cold regions (Park *et al.* 2019). For high‐elevation and high‐latitude vegetation, increasing evidence from satellite observations indicate that over the past decades of warming, spring phenology (Badeck *et al.* 2004; Shen *et al.* 2015a) and the timing of maximum photosynthesis (Xu *et al.* 2016; Park *et al.* 2019) have tended to advance, while autumn phenology has tended to be delayed (Barichivich *et al.* 2013; Liu *et al.* 2016). However, despite the abundant evidence from remote sensing, there is a dearth of corresponding field evidence on long‐term phenological changes in these ecosystems, as well as the underlying mechanisms driving phenological changes (if any).

**Figure 1:**
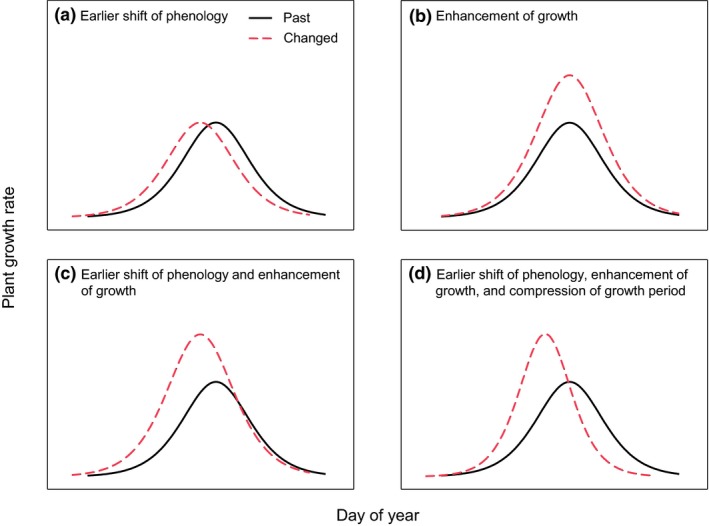
Conceptual representation of the mechanisms of the advancement in spring phenology under climate warming. Four potential scenarios responsible for this advancement are presented, including earlier shift of phenological pattern (a), enhancement of growth under the same phenological pattern (b), both earlier shift of phenology and enhancement of growth (c), and earlier shift of phenology, enhancement of growth, and compression of growth period (d).

Warming can influence plant phenology by shifting the timing of phenological events, changing growth rate, or both (Buitenwerf *et al.* 2015; Gonsamo *et al.* 2018). A number of studies have documented that warming advances the phenology of plants in the spring by accelerating the ecodormancy break, but delays spring phenology by slowing down the endodormancy break (Bibi *et al.* 2018; Piao *et al.* 2019). Warming can also advance autumn phenology directly or indirectly by reducing soil water availability (Estiarte & Peñuelas 2015; Liu *et al.* 2016). Furthermore, warming often enhances plant growth because air temperatures are commonly lower than the optimal temperature for plant growth (Lambers & Chapin 1998; Gonsamo *et al.* 2018). On the other hand, warming may reduce plant growth if the warming‐induced extension of growing season increases the risk of spring frost damage (Richardson *et al.* 2018; Liu *et al.* 2018b). In addition, the effect of warming on phenology and growth rate can be modulated by shifts in plant community and functional group composition (Meng *et al.* 2017; Wolf *et al.* 2017), a phenomenon more readily observed in long‐term studies (Hudson & Henry 2009; Harte *et al.* 2015). However, it remains unclear whether the long‐term phenological changes detected by remote sensing result from shift in the timing of phenological events (Fig. 1a) and/or change in growth rate (Fig. 1b) in response to climate warming.

Here, we report on a unique long‐term (1980–2014) record of seasonal biomass dynamics and community composition in an alpine grassland on the Tibetan Plateau, which has experienced a warming trend doubling the rate of the global average over the past 50 years (Hansen *et al.* 2010; Chen *et al.* 2013). Using this dataset, we aimed to uncover how the observed climate change affects growth patterns of alpine plants hidden behind the acknowledged advancement in spring phenology. Specifically, we tested the hypothesis (1) that climate warming would enhance vegetation growth in addition to promoting an earlier shift in phenology (Fig. 1c), as warming can alleviate the constraints of low temperature on both plant ecodormancy break and growth rate. We further tested the hypothesis (2) that warming would also shorten the vegetation growth period (Fig. 1d), which can cancel out the effect of enhanced growth rate on biomass production. This is because our previous study documented no systematic changes in annual biomass production in the alpine grassland (Liu *et al.* 2018a). We finally tested the hypothesis (3) that a shift in functional group composition would play an important role in reshaping vegetation growth patterns under climate warming, as climate warming often shifts plant community composition and differentially influences plant phenology at the species level (Dorji *et al.* 2013; Meng *et al.* 2017; Suonan *et al.* 2017).

## Materials and methods

### Site description

We conducted this study at the Haibei National Alpine Grassland Ecosystem Research Station (37°36′ N, 101°19′ E, 3215 meters above sea level) located in the northeastern part of the Tibetan Plateau, in Qinghai Province, China (Fig. S1). The climate of the study site is influenced by a continental monsoon and characterized by short, cool summers and long, cold winters. From 1981 to 2014, average annual air temperature at the station was −1.1 °C, and monthly mean air temperature ranged from −14.42 to 10.46 °C. The highest temperature occurred in July and the lowest temperature occurred in January. Average annual precipitation over the three decades was 487.8 mm, with most annual precipitation (84%) falling from May to September. The area is covered by mesic meadow consisting of C_3_ perennial species, dominated by grasses, such as *Stipa aliena*, *Elymus nutans* and *Helictotrichon tibeticum*, mixed with forbs, including *Gentiana straminea*, *Tibetia himalaica*, *Saussurea pulchra* and *Medicago ruthenica*, and sedges such as *Kobresia humilis* and *Carex przewalskii* (Ma *et al.* 2017). The soil is classified as Mat‐Gryic Cambisol in Chinese Soil Taxonomy and as borolls in US Soil Taxonomy. In the 0–10 cm soil layer, soil bulk density is 0.8 g cm^−3^, soil organic carbon content is 63.1 g kg^−1^, and soil pH is 7.8 (Liu *et al.* 2018a). Following local practice, the site has been lightly grazed as winter pasture since 1980.

### Long‐term monitoring of annual biomass production and seasonal biomass dynamics

From 1980 to 2014, annual biomass production of the plant community was monitored using a harvesting method. For our study, we defined annual biomass production as the maximum aboveground biomass observed in August or September (Liu *et al.* 2018a). Annual biomass production was further separated into grass, forb and sedge functional groups in the following years: 1980–1985, 1989, 1998–2000 and 2006–2014. Seasonal biomass dynamics of the plant community were monitored by clipping aboveground biomass once or twice each month from May to September during the periods of 1980–1985, 1989, 2002–2004, 2006–2010 and 2012–2014; the seasonal biomass dynamics of different plant functional groups were further monitored during 1980–1983 and 2007–2010. After harvesting, live plant samples were oven‐dried at 65 °C until they reached a constant weight.

Two sampling methods were used from 1980 to 2014 to monitor plant biomass (Liu *et al.* 2018a). Before 2005, five to ten 50 × 50 cm quadrats were randomly clipped during each harvest within a permanent 250 × 230 m area. Starting in 2005, a new strategy of systematic sampling was adopted. An area of 150 × 150 m was divided into 25 permanent squares, and the five squares on the diagonal were chosen. Each chosen square was further divided into 25 blocks that were each 6 × 6 m. Five 25 × 25 cm replicates were randomly harvested from one of the 25 blocks in five chosen squares.

### Parameters to describe plant fast‐growing phase

Seasonal biomass dynamics were simulated using linear, exponential, monomolecular and logistic functions (Paine *et al.* 2012). We found that a three‐parameter logistic function appropriately described the aboveground biomass dynamics across growing seasons (Fig. S2):

$$AGB=\frac{L}{1\;+\;e^{-k(x-x_{0})}}$$

where *AGB* is the aboveground biomass and *x* is the Julian day. The parameters *L*, *k* and *x*_0_ represent the annual maximum aboveground biomass, the intrinsic rate of plant growth and the timing of maximum growth, respectively (Table S1). Fitted results from this method were validated with annual aboveground biomass production data (*r* ^2^ = 0.84, *P* < 0.001). We then calculated the growth rate for each day by using differential coefficients from this fitted equation (Fig. S3). Finally, we defined spring, summer and autumn biomass production as the sum of daily growth rate from April to May, from June to July and from August to September, respectively (Zhang *et al.* 2013a).

To explore how changes in plant phenology and growth rate influenced seasonal biomass production over time, we used the fast‐growing phase concept (Gregorczyk 1991). The mid‐season ‘fast‐growth phase’ was identified by the seasonal dynamics of growth rate (Fig. S3). Specifically, the start and end of the fast‐growing phase were defined as the days of maximum increase and maximum decrease in growth rate, which also correspond to the days at which aboveground biomass reaches 21% and 79% of the annual maximum biomass, respectively. The length of the fast‐growing phase was calculated as the number of days between the start and end of the fast‐growing phase.

### Statistical analysis

We conducted linear regression to examine long‐term interannual trends of environmental factors (air temperature, precipitation, humidity index, and soil moisture) and annual biomass production. Although we had no observational data of seasonal biomass dynamics from 1990 to 2001, plant phenology as reflected by the Normalized Difference Vegetation Index (NDVI) data showed linear trends of change over time (Fig. S4; Supplementary Information section S1 and S2). We thus used linear regression to analyse long‐term interannual trends of seasonal biomass production (spring production, summer production and autumn production), phenology (start, end, and length of the fast‐growing phase or growing season and timing of maximum growth), and rate of maximum growth. We calculated the interannual rate of change using the slope of linear regression. We used *t*‐tests to test for differences in relative abundance of functional groups and in their phenology and growth rate between 1980 to 1983 and 2007 to 2010. We used Pearson’s correlation coefficients to investigate linkages between seasonal biomass production, phenology and growth rate over time.

To investigate how air temperature and precipitation influenced plant phenology (start and end of the fast‐growing phase and timing of maximum growth), we developed 15 linear models within different temporal periods. Besides the monthly values (January to August), we split the air temperature and precipitation data into four phases corresponding with stages of plant growth and their three combinations consisting of the adjacent two phases. Precipitation from the previous year was retained for plant growth in the following year (Robinson *et al.* 2013), and October to December in the previous year was defined as the ‘dormant period’. Most of the species at our site begin to grow in April (Zhou *et al.* 2014), so we labelled the period between January and March as the ‘pre‐growing season’. Due to low growth rates in April and May, we labelled this period of growth as the ‘early growing season’. The period from June to August, when plants grow rapidly, was labelled as the ‘mid‐growing season’. The month of September was not included in this analysis not only because it lagged behind the fast‐growing phase, but because September precipitation was not usually retained in the following year. To assess climatic control of the rate of maximum vegetation growth, we developed four linear models within the periods of June, July, August, and the ‘mid‐growing season’ when climatic conditions directly influenced vigorous growth rate. Model performance was evaluated using Akaike’s information criterion (AIC). Models were considered to have statistical support if *P* < 0.05.

We used linear regression to investigate the relationship between the start of the fast‐growing season and pre‐ and early‐season growing degree days over time. The growing degree day requirement for vigorous plant growth was calculated as an integration of daily average air temperature above a threshold of 0 °C from January 1 to May 31 (Fu *et al.* 2019). In addition, we used linear regression to explore the relationship between the end of the fast‐growing season and mid‐growing season soil moisture over time. We also used general linear mixed‐effects models to evaluate the effects of air temperature, precipitation and aboveground plant biomass on monthly mean soil moisture during mid‐growing season (June to August), in which month was treated as a random factor (‘lme4’ package in R software). To quantify the relative importance of the three predictor variables on soil moisture, we next performed a multimodel inference based on AIC (Deng *et al.* 2018). Specifically, we first used the ‘lmer’ function to fit a global model, and then used the ‘dredge’ function to generate a full submodel set from the global model (‘MuMIn’ package in R software). Finally, we produced a top model set based on a cut‐off of ΔAIC < 5, and used the ‘model.avg’ function to estimate the model parameters of the top model.

To compare the differences in changes in phenology and growth rate of different functional groups over time, we standardized growth patterns for grass, forb and sedge functional groups by dividing them by their respective annual biomass production. We also used partial redundancy analyses to quantify the relative importance of the differential changes in phenology and growth rate of functional groups and the shifts in functional group composition for explaining variance in community growth patterns (‘vegan’ packages in R software). All statistical analyses were conducted using R 3.5.0 software (R Core Team, 2018).

## Results

### Long‐term changes in annual and seasonal biomass production

**Figure 2:**
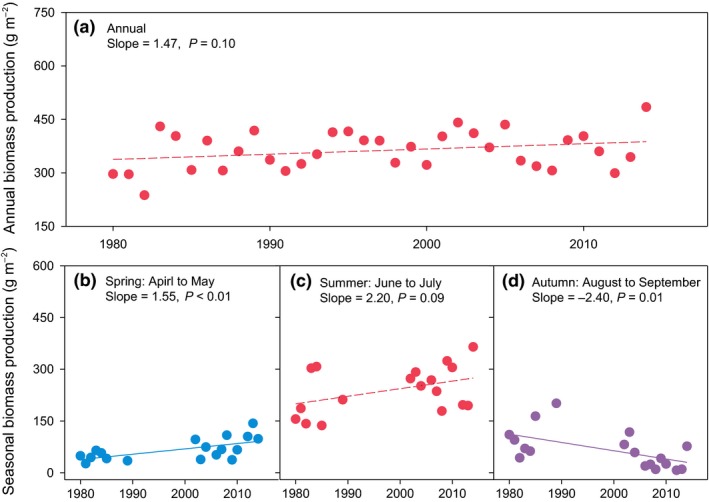
Long‐term (1980–2014) changes in annual and seasonal aboveground biomass production at Haibei research station. The temporal trends in annual (a), spring (b), summer (c) and autumn (d) biomass production. Solid and dashed regression lines indicate statistically significant and non‐significant trends at the 0.05 level, respectively.

Over the past 35 years, annual biomass production ranged from 237.3 to 484.5 g m^−2^ year^−1^, without exhibiting any significant overall trend (Fig. 2a; an increase of 14.7 g m^−2^ per decade; *r* ^2^ = 0.08, *P* = 0.10). However, seasonal biomass production showed strikingly different patterns: spring production (April–May) increased by 15.5 g m^−2^ per decade (Fig. 2b; *r* ^2^ = 0.39, *P* < 0.01), autumn production (August–September) decreased by 24.0 g m^−2^ per decade (Fig. 2d; *r* ^2^ = 0.32, *P* = 0.01), whereas summer production (June–July) did not exhibit any significant trend (Fig. 2c; an increase of 22.0 g m^−2^ per decade; *r* ^2^ = 0.17, *P* = 0.09).

### Long‐term changes in community phenology and growth rate

**Figure 3:**
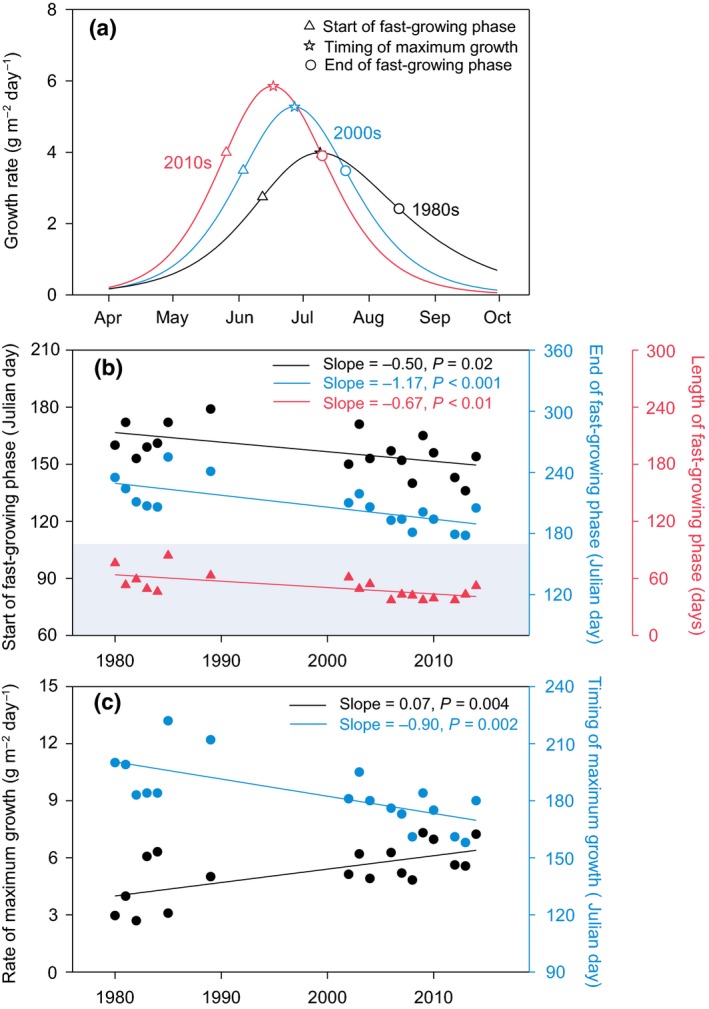
Long‐term (1980–2014) changes in plant growth patterns. (a) Data are mean values in 1980s (*n* = 7 years), 2000s (*n* = 7 years) and 2010s (*n* = 4 years). (b) The temporal trends in the start, end and length of fast‐growing phase. (c) The temporal trends in rate and timing of maximum growth. Solid regression lines indicate statistically significant trends at the 0.05 level.

From 1980 to 2014, the start of the fast‐growing phase advanced at a rate of 5 days per decade (Fig. 3a and b; *r* ^2^ = 0.31, *P* = 0.02), while the end of the fast‐growing phase advanced at a rate of 12 days per decade (*r* ^2^ = 0.51, *P* < 0.001). The length of the fast‐growing phase thus became shorter over time (7 days per decade; *r* ^2^ = 0.42, *P* < 0.01). Over the same period, the rate of maximum growth increased by 0.7 g m^−2^ day^−1^ per decade (Fig. 3c; *r* ^2^ = 0.41, *P* = 0.004), and the timing of maximum growth advanced at a rate of 9 days per decade (*r* ^2^ = 0.47, *P* = 0.002). These changes in growth patterns were observed in years that had more frequent measurements (Fig. S5; ≥7 times per year). Further analysis showed that the earlier phenology of the fast‐growing phase was related to increased spring production and reduced autumn production, whereas the shorter fast‐growing phase and the enhanced maximum growth jointly led to no change in summer production (Fig. S6).

### Effects of climate change on community phenology and growth rate

**Figure 4:**
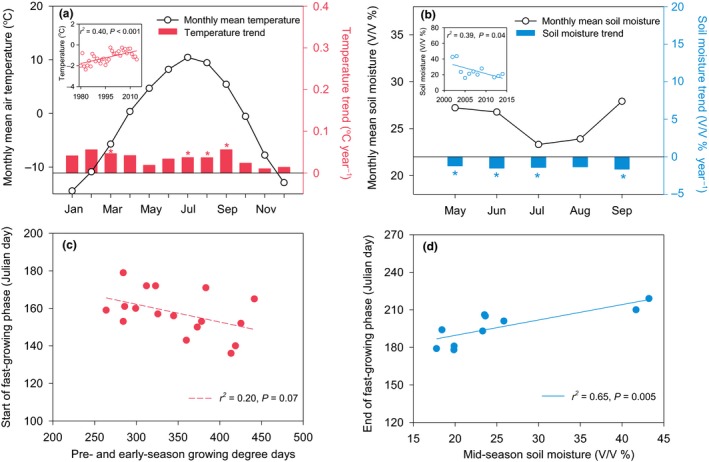
Controls of air temperature on the start of the fast‐growing phase and soil moisture on the end of the fast‐growing phase. Seasonal dynamics in monthly mean air temperature (a) and soil moisture at the 5 cm depth (b) and their changing trends. Lines with circles indicate monthly mean values; bars indicate their changing rates, as expressed by the slopes of linear regressions between years and monthly averages, and * indicates statistically significant at *P* < 0.05. The insets in (a) and (b) indicate the interannual trends in annual mean air temperature and soil moisture, respectively. Relationships between pre‐ and early‐season growing degree days (January–May) and the start of the fast‐growing phase (c) and between mid‐season soil moisture (June–August) and the end of the fast‐growing phase (d). Solid and dashed regression lines indicate statistically significant and non‐significant trends at the 0.05 level, respectively.

Over 35 years, annual mean air temperature at the study site increased by 0.4 °C per decade (Fig. 4a; *r* ^2^ = 0.40, *P* < 0.001), and the warming trend was statistically significant (*P* < 0.05) for both March and from July to September. Annual precipitation did not vary systematically (Fig. S7a; *r* ^2^ = 0.06, *P* = 0.16); precipitation in July, however, decreased by 11.0 mm per decade (*r* ^2^ = 0.13, *P* = 0.04). In contrast, the annual humidity index tended to decline (Fig. S7b; *r* ^2^ = 0.11, *P* = 0.06), with a significant decline in July (*P* = 0.02). In addition, soil moisture at the 5 cm depth decreased from 2002 to 2014 (Fig. 4b; *r* ^2^ = 0.39, *P* = 0.04). Overall, the site became both warmer and drier over our study period.

Increased pre‐ and early‐season growing degree days (January–May) and warmer March temperatures were associated with an earlier start of the fast‐growing phase (Fig. 4c and Fig. S8), while a reduction in both soil moisture and precipitation during mid‐growing season (June–August) was related to an earlier end of the fast‐growing phase (Fig. 4d and Fig. S8). At the same time, increased temperatures and reduced precipitation in July were related to a higher rate of maximum growth (Fig. S8).

### Changes in abundance, phenology and growth rate of different functional groups

**Figure 5:**
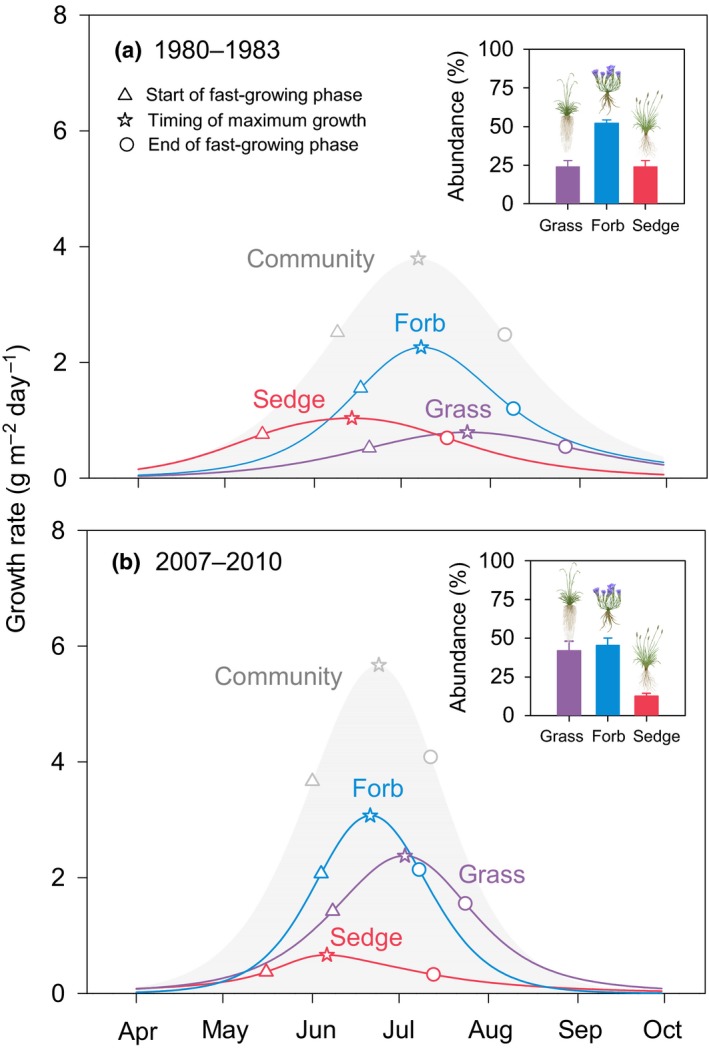
Comparisons of growth patterns of different plant functional groups. Data are mean values during 1980–1983 (a) and 2007–2010 (b). The insets in (a) and (b) indicate relative abundance of different functional groups.

Between the two periods, 1980–1983 and 2007–2010, the abundance of grasses increased and the abundance of forbs and sedges decreased (Fig. 5). Over the same period, grasses and forbs were more sensitive to climate change than sedges (Fig. S9). Specifically, grasses and forbs started and ended growth earlier, had a higher growth rate, but had a shorter fast‐growing phase (Fig. S10). In contrast, sedges did not exhibit any significant trends. Partial redundancy analysis further showed that the changes in growth patterns of grasses and forbs, rather than the shifts in plant functional group composition, were mainly responsible for the observed changes in community phenology and growth rate (Fig. S11).

## Discussion

**Figure 6:**
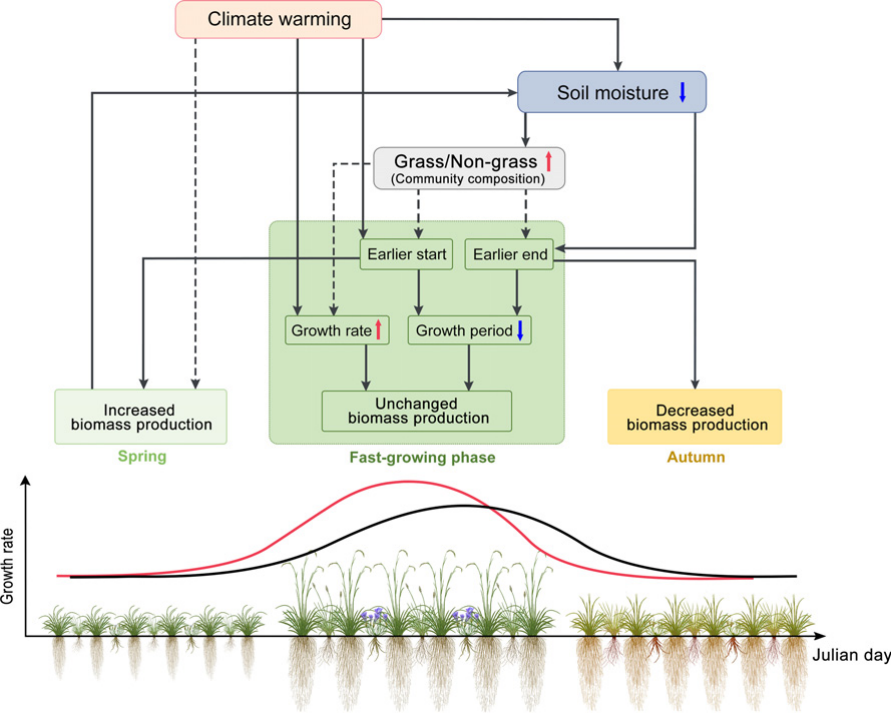
Illustration of mechanisms of long‐term changes in plant growth patterns in a Tibetan alpine grassland. Climate warming shifted the start of fast‐growing phase earlier and enhanced rate of maximum growth while reduction in mid‐season soil moisture accelerated the end of fast‐growing phase. In contrast, a shift in functional group composition towards grasses induced by reduced soil moisture contributed less to community phenology and growth rate (black dotted arrows in Figure). The positive effect of enhanced maximum growth on biomass production of fast‐growing phase was cancelled out by the negative effect of shortened growth period. The earlier phenology led to an increase in spring biomass production and a decrease in autumn biomass production. The increased spring biomass production contributed to reduced mid‐season soil moisture due to more water demand of plant growth.

Our results support the first two hypotheses that long‐term climate warming enhanced plant maximum growth and shortened the fast‐growing phase, in addition to shifting phenology earlier. These changes in growth patterns led to altered seasonal biomass production: spring production increased, summer production remained relatively constant and autumn production decreased over time in this alpine grassland (Fig. 6). Inconsistent with our third hypothesis, the observed changes in growth patterns were largely attributed to changes in phenology and growth rate of grasses and forbs, rather than effects of shifting functional group composition. Altogether, this study, to our knowledge, provides the first *in situ* evidence that the growth patterns of alpine grassland plants have strongly responded to long‐term climate change, despite the lack of systematic change in annual biomass production.

### Earlier phenology and faster growth jointly contributed to changes in growth patterns

Results from 35 years of monitoring show an earlier start of the fast‐growing phase and enhancement in maximum growth in the alpine grassland we studied. These changes indicate that earlier phenology and enhanced growth in spring jointly contributed to the advancement in the start of the growing season detected by satellite‐derived NDVI data (see Fig. S4a). The earlier start of the fast‐growing phase was associated with increases in spring temperatures and growing degree days, which may accelerate ecodormancy break and spring snow thaw (Chen *et al.* 2015; Suonan *et al.* 2017; Bibi *et al.* 2018).

In contrast, enhanced maximum growth during the mid‐growing season may be attributable to three factors. First, climate change led to increased synchronization in the timing of maximum growth of different functional groups (see Fig. S10c). Second, warming increased maximum growth of grasses and sedges, likely because mid‐growing season temperatures were still lower than their optimal growth temperature (Lambers & Chapin 1998). Third, declining mid‐season precipitation might have also contributed to increased plant growth, as cloud cover accompanying frequent mid‐season precipitation events tends to reduce light availability to plants (Graham *et al.* 2003; Piao 2003). Overall, an earlier start of the fast‐growing phase and enhancement in maximum growth suggest that climate warming benefits early season plant growth in the alpine grassland community.

Our analyses also indicate that the end of the fast‐growing phase advanced more than the start of the fast‐growing phase. One possible explanation for the earlier end is that climate change may have led to increased plant water stress during the middle of the growing season (Ernakovich *et al.* 2014; Estiarte & Peñuelas 2015). Consistent with this mechanism, we found a reduction in mid‐season soil moisture over our study period, which was attributed to (1) the reduction in mid‐season precipitation, and (2) greater consumption of available soil water by the warming‐induced higher spring biomass (Table S2). Admittedly, our consideration of this mechanism does not rule out other non‐mutually exclusive mechanisms. For example, the length of the plant growth period may be controlled by intrinsic processes such as programmed cell death (Lim *et al.* 2007; Steltzer & Post 2009), which may cause an earlier end of the plant growth following an earlier start. Furthermore, a reduction in pre‐season soil moisture under warming may have contributed to the advance in the end of fast growth (Yang *et al.* 2019). Further research is needed to investigate these potential mechanisms.

### Minor contribution of functional group composition shift to changes in growth patterns

Previous work suggests that shifts in functional group composition influence phenological response to temperature change in Tibetan alpine grasslands (Meng *et al.* 2017). Our findings reinforce this idea, but reveal that shifts in functional group composition had limited effects on long‐term changes in plant growth patterns in this alpine grassland. Our previous study, at the same site, suggested that under a warmer and drier climate, alpine plant community composition shifts towards deep‐rooted grasses at the expense of the shallow‐rooted sedges and forbs (Liu *et al.* 2018a). In the present study, we found that the fast growth period for grasses occurred later than for both forbs and sedges, and that forbs had the highest maximum growth rate among all three functional groups (see Fig. S9). Thus, the observed shifts in functional group composition would have led to a delayed phenology and a reduced maximum growth if it is important; however, this is not what we observed.

It should be noted that sedges were less sensitive to climate change than were grasses and forbs. The start of sedge growth did not obviously change in response to warmer spring temperatures – a pattern supported by a warming experiment at the same study site (Suonan *et al.* 2017). One potential explanation is that photoperiod plays a modulating role and inhibits the positive effect of climate warming on the start of sedge growth (Keller & Körner 2003; Körner & Basler 2010). In contrast, no significant change in the end of sedge growth may be attributed to the lack of effect of mid‐season soil water reduction on sedge water stress, as sedges nearly complete their fast‐growing phase in June when soil moisture is relatively high.

In agreement with the changes in alpine grassland growth patterns we observed, many studies of crop phenology find that a warmer climate shifts sowing timing earlier and enhances crop growth (Patil *et al.* 2010; Sacks & Kucharik 2011; Liu *et al.* 2013; Zhang *et al.* 2013b); however, the mechanisms between crop and alpine systems might differ. Compared with relatively simple cropland communities, growth in natural grassland depends on diverse responses of different species to warming, influenced by shifts in functional group composition. In addition, phenology in perennial grassland, such as our alpine grassland, may be influenced by winter warming through slowing down endodormancy break. In contrast, the warming effect does not always occur in croplands because most crops complete their life cycle within one year under often intense human management.

Our findings highlight a crucial contribution of climate change to long‐term changes in grassland growth patterns in the temperature‐sensitive region. However, we cannot rule out the potential roles of other factors, such as increasing nitrogen deposition or ecological succession, in driving the observed patterns. The relative contributions of these drivers to plant growth patterns warrant future investigation. Despite this caveat, this study has several important implications for understanding ecosystem function and vegetation‐climate feedbacks. First, the earlier plant phenology might alter the life cycle of alpine plants via effects on pollination and autumn seed maturation. Second, the earlier plant phenology could cause trophic decoupling of food webs if phenological shifts of other trophic levels cannot keep pace with changes in plant phenology (Post *et al.* 2008; Thackeray *et al.* 2016). Third, the changes in growth patterns of the aboveground plant component may influence belowground phenology because root growth depends on leaf photosynthesis in grasslands (Steinaker & Wilson 2008). Lastly, the earlier phenology and faster growth of plants may generate a cooling effect through vegetation‐evapotranspiration feedback in the alpine region (Jeong *et al.* 2009; Shen *et al.* 2015b).

In summary, based on a unique 35‐year dataset of seasonal biomass dynamics, we found that climate change reshaped growth patterns of alpine plant communities by shifting phenology earlier, enhancing growth rate and shortening growth period. This finding improves our understanding on the mechanism underlying the advancement in alpine spring phenology under climate warming. Furthermore, we found that the increase in spring production due to the earlier start of fast‐growing phase was counteracted by the reduction in autumn production due to an earlier end, contributing to no change in annual biomass production. Thus, elucidating how phenological shifts differentially affect biomass production during different growth stages holds the key to better understanding the responses of grassland biomass production to future climate warming.

## Authorship

J.‐S.H. designed the research. H.W., H.Y.L. and X.Z. compiled and analysed the data. H.W., H.Y.L and J.‐S.H wrote the first draft. G.M.C., Y.K.L. and F.W.Z. supported the collection of monitoring data. All authors contributed to the writing and discussion of the paper.

## Data Availability

Data are archived as.csv files in the Figshare digital repository: https://doi.org/10.6084/m9.figshare.11663997
