# Supplementary material for: Alpine grassland plants grow earlier and faster but biomass remains unchanged over 35 years of climate change
Source: Ecol Lett. 2020 Feb 12;23(4):701–10. doi: 10.1111/ele.13474 (PMC7154776; doi:10.1111/ele.13474)
Supplement: Supplementary file 1 [file ELE-23-701-s001.docx]

Alpine grassland plants grow earlier and faster but biomass remains unchanged over 35 years of climate change

***Supplementary Materials***

**S1 Supplementary Methods**

**S2 Supplementary Results**

**Supplementary Figures: 11**

**Supplementary Tables: 2**

**Supplementary References**

**S1 Supplementary Methods**

***Extracting data from Normalized Difference Vegetation Index (NDVI) products***

The third generation Global Inventory Monitoring and Modeling System NDVI Index (GIMMS NDVI_3g_) dataset was used to estimate the start, end, and length of growing season at this study site. The dataset spanning from 1982 to 2014 was derived from the Advanced Very High Resolution Radiometer (AVHRR) sensors, with a 15-day temporal frequency and an 8-km spatial resolution (http://ecocast.arc.nasa.gov/data/pub/gimms/3g.v1/). Maximum composite method was used to minimize the effects of atmospheric conditions, such as cloud cover, aerosols, and atmospheric vapor (Pinzon & Tucker 2014).

The Savitzky and Golay smoothing filter was used to perform data filtering and to reconstruct time series of NDVI (Zhao *et al.* 2015). Winter NDVI values were generally negatively biased by the impact of snow cover, and thus were replaced by the mean NDVI between the end of the growing season (late October) and the beginning of winter snow cover (late November) (Beck *et al.* 2006). By fitting a double logistic function to the smoothed and snow-corrected NDVI time series, we defined the start and end of growing season as the days when the rate of change in NDVI curvature reached the first local maximum value and the last local minimum value, respectively (Busetto *et al.* 2010). The length of growing season was calculated as the number of days between start and end of growing season. The timing of maximum increase of NDVI was defined the day-of-year when the double logistic curve first reached its half-maximum value (Busetto *et al.* 2010).

**S2 Supplementary Results**

***Long-term changes in phenology and growth rate based on NDVI data***

From 1982 to 2014, the start of the growing season advanced at a rate of 2 days per decade (Fig. S4a; *r^2^* = 0.13, *P* = 0.04), while the end of the growing season delayed at a rate of 2 days per decade (*r^2^* = 0.29, *P* = 0.001). As a result, the growing season lengthened by 4 days per decade (*r^2^* = 0.28, *P* = 0.002). Over time, the NDVI at the timing of maximum increase showed no significant trend (Fig. S4b; *r^2^* = 0.03, *P* = 0.30), but the timing of maximum increase of NDVI significantly advanced at a rate of 2 days per decade (*r^2^* = 0.14, *P* = 0.03).

**Supplementary Figures**

**
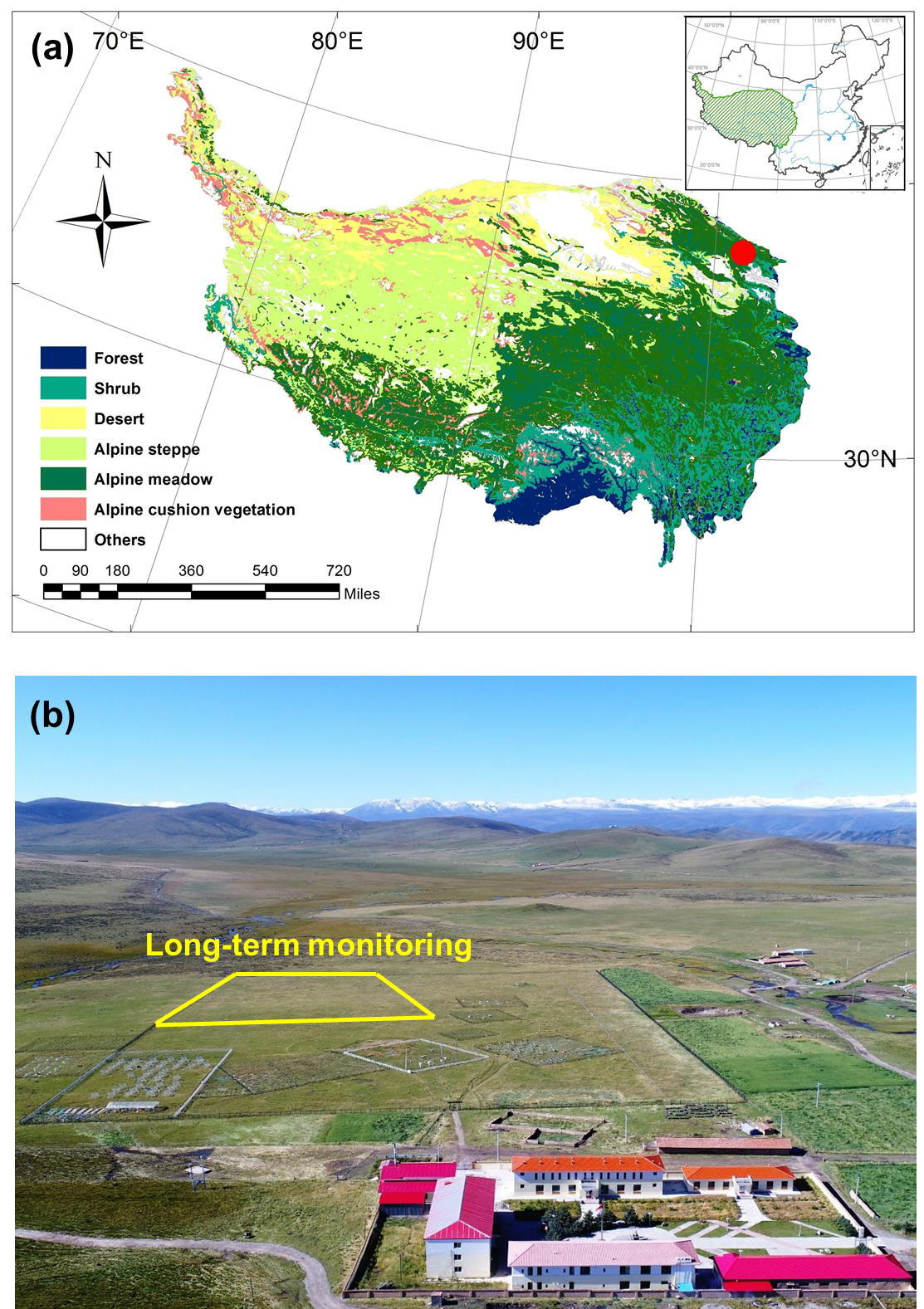
**

**Figure S1** Location and landscape of the study site. (a) Geographical location of the study site on the Tibetan Plateau. (b) Landscape of the long-term monitoring area, located at Haibei National Alpine Grassland Ecosystem Research Station.

**Figure S2** Aboveground biomass dynamics for the whole community from 1980 to 2014. The process was monitored by clipping aboveground biomass once or twice each month in the growing season, and fitted by the logistic growth curves.

**Figure S3** Schematic diagram of the concept of the fast-growing phase. (a) A sketch map describing aboveground biomass dynamics. (b) A sketch map describing plant growth rate. t_1_, t_3_, and t_2_ refer to the start and end of fast-growing phase and the timing of maximum growth, respectively. y_1_ (y_1_’), y_3_ (y_3_’), and y_2_ (y_2_’) indicate the corresponding aboveground biomass (growth rate) when the start and end of fast-growing phase and maximum growth occur, respectively.

**Figure S4** Long-term (1982–2014) changes in plant phenology derived from satellite-derived NDVI data and in NDVI at the timing of maximum increase. (a) The temporal trends in the start, end, and length of growing season. (b) The temporal trends in NDVI at the timing of maximum increase and timing of maximum increase of NDVI. Solid and dashed regression lines indicate statistically significant and non-significant trends at the 0.05 level, respectively.

**Figure S5** Comparisons of plant community phenology and growth rate between 1980s and 2010s. Data are mean values ± 1 standard error (n = 3 years) for the start, end (a), and length (b) of fast-growing phase, and timing (c) and rate (d) of maximum growth. Note that sampling frequency of the selected years was more than 7 times. Letters (a and b) indicate statistically significant difference at the 0.05 level.

**
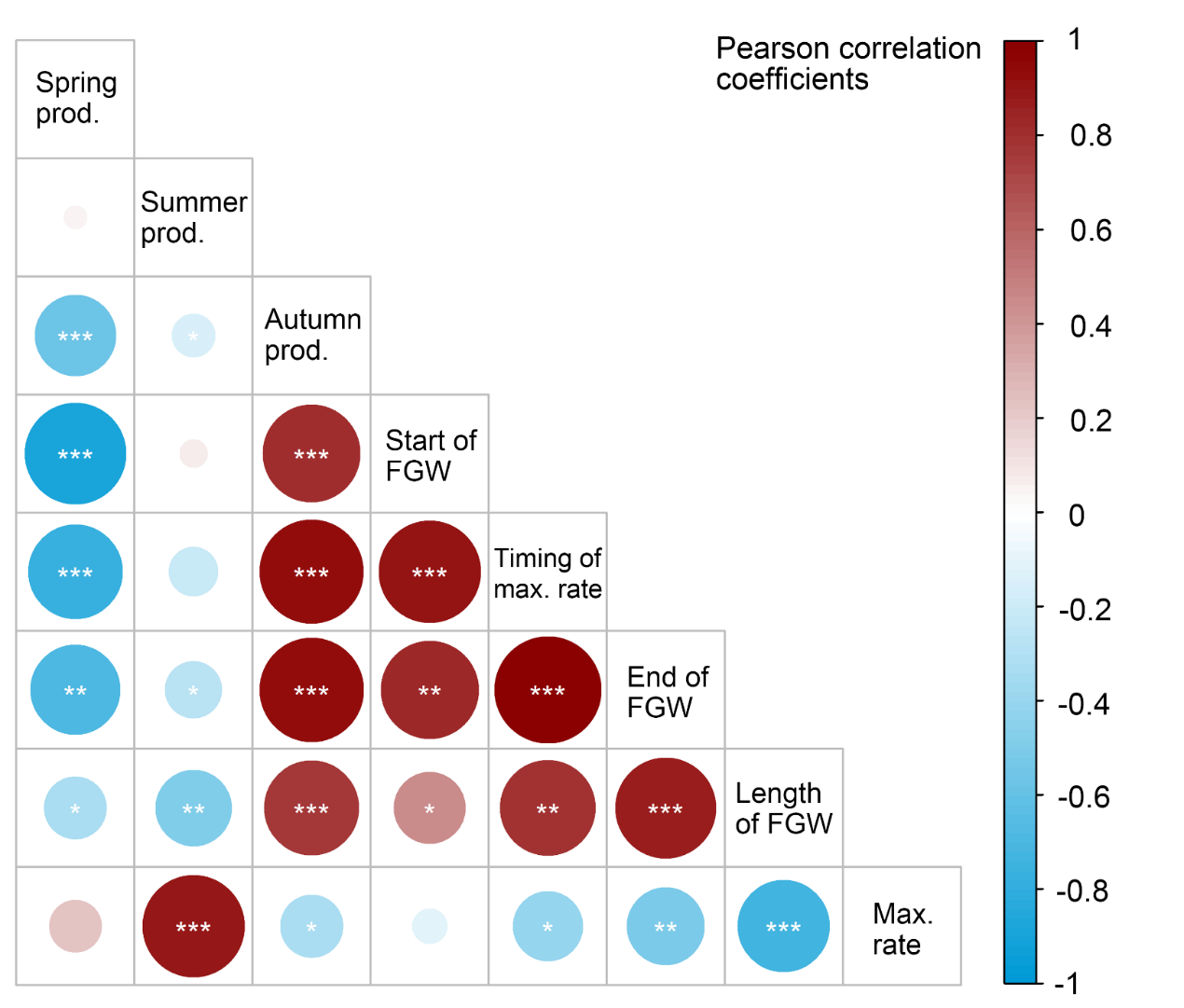
**

**Figure S6** Pearson correlation coefficients between seasonal biomass production (spring production, summer production, and autumn production), phenology (start, end, and length of fast-growing phase, and timing of maximum growth), and rate of maximum growth. The color scale and the circle size indicate the magnitude of correlation coefficients. *, **, and *** indicate statistically significant at *P* < 0.05, *P* < 0.01, and *P* < 0.001, respectively. Prod., production; Max. rate, maximum growth rate; FGW, fast-growing phase.

**Figure S7** Long-term changes in precipitation and humidity index at Haibei research station. Seasonal dynamics in monthly mean precipitation (a) and humidity index (b) and their changing trends. Lines with circles indicate monthly mean values; bars indicate their changing rates, as expressed by the slopes of linear regressions between years and monthly averages, and * indicates statistically significant at *P* < 0.05. The insets in (a) and (b) indicate the interannual trends in annual mean precipitation and humidity index, respectively. Dashed regression lines indicate statistically non-significant trends at the 0.05 level. Note that humidity index was defined as the ratio of annual precipitation to potential evapotranspiration, and the potential evapotranspiration was estimated using Romanenko method.

**Figure S8** Controls of air temperature and precipitation on the standardized plant community (comm.) phenology (start and end of fast-growing phase and timing of maximum growth) and growth rate (rate of maximum growth) during 1980–2014 (n = 17 years). October to December in pre-year was labeled as the dormant period (Dorm), January to March as the pre-growing season (Pre), April and May as the early growing season (Early), and June to August as the mid-growing season (Mid). The circles were shown when the correlation is statistically significant at the 0.05 level.

**Figure S9** Comparisons of the standardized growth pattern of different plant functional groups. Data are mean values for different functional groups during 1980–1983 (a) and 2007–2010 (b). The shaded area refers to the standardized growth pattern of the whole community.

**Figure S10** Comparisons of plant phenology and growth rate between 1980–1983 and 2007–2010. Data are mean values ± 1 standard error (n = 4 years) for the start, end (a), and length (b) of fast-growing phase, and timing (c) and rate (d) of maximum growth derived from the standardized growth patterns of each functional group and the whole community. G, Grass; F, Forb; S, Sedge; Comm., Community. ^, *, and ** indicate statistically significant difference at *P* < 0.1, *P* < 0.05, and *P* < 0.01, respectively.


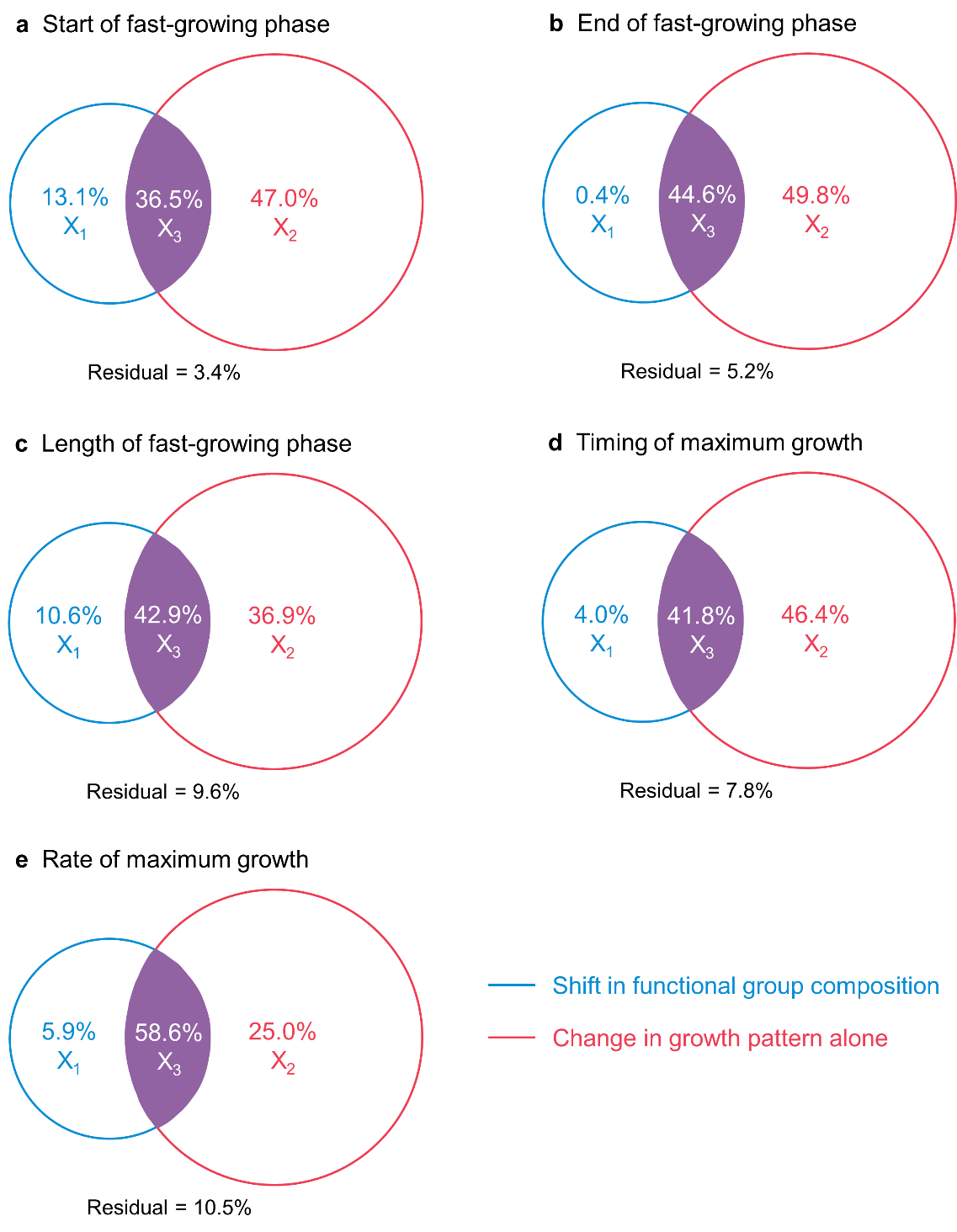


**Figure S11** Partial redundancy analysis showing the percentages of variance in community phenology and growth rate during the fast-growing phase explained by four categories: pure effect of shift in functional group composition (X_1_), pure effect of change in growth patterns of different functional groups (X_2_), joint effect of shift in functional group composition and their changes in growth patterns (X_3_), and residual variation. (a) Start of fast-growing phase. (b) End of fast-growing phase. (c) Length of fast-growing phase. (d) Timing of maximum growth rate. (e) Rate of maximum growth.

**Supplementary Tables**

**Table S1.** Summary of parameters of the logistic growth curve$(y=\frac{L}{1+e^{-k\left( x-x_{0} \right)}})$ simulating aboveground biomass dynamics for the whole community during 1980–2014.

| **Years** | **Annual peak biomass (*L*)** | **Intrinsic growth rate (*k*)** | **Timing of maximum growth (*x_0_*)** | ***r^2^*** |
| --- | --- | --- | --- | --- |
| 1980 | 348.8 | 0.034 | 200 | 0.99 |
| 1981 | 315.1 | 0.051 | 198 | 0.98 |
| 1982 | 236.1 | 0.046 | 182 | 0.94 |
| 1983 | 443.4 | 0.055 | 183 | 1.00 |
| 1984 | 430.5 | 0.059 | 183 | 1.00 |
| 1985 | 430.1 | 0.029 | 222 | 1.00 |
| 1989 | 484.3 | 0.041 | 212 | 1.00 |
| 2002 | 467.0 | 0.044 | 180 | 1.00 |
| 2003 | 454.9 | 0.055 | 195 | 1.00 |
| 2004 | 392.0 | 0.050 | 180 | 1.00 |
| 2006 | 340.0 | 0.074 | 175 | 1.00 |
| 2007 | 329.7 | 0.063 | 173 | 1.00 |
| 2008 | 300.4 | 0.064 | 160 | 0.98 |
| 2009 | 403.4 | 0.073 | 183 | 0.98 |
| 2010 | 398.0 | 0.070 | 175 | 0.99 |
| 2012 | 309.8 | 0.073 | 161 | 1.00 |
| 2013 | 352.7 | 0.063 | 157 | 1.00 |
| 2014 | 548.2 | 0.053 | 180 | 1.00 |

**Table S2.** Summarized results of general linear mixed-effects model on the effects of air temperature, precipitation, and plant biomass on mid-season soil moisture (June to August) from 2002 to 2014.

| **Parameter** |  | **Estimate (CI)** | ***P*-value** | **Relative importance** |
| --- | --- | --- | --- | --- |
| Intercept |  | 3.16 (3.03, 3.29) | <0.001 |  |
| Air temperature |  | -0.14 (-0.43, 0.15) | 0.35 | 0.09 |
| Precipitation |  | 0.31 (0.07, 0.56) | 0.01 | 0.64 |
| Plant biomass |  | -0.24 (-0.48, 0.003) | 0.05 | 0.32 |

The table represents the standardized parameter estimates, 95% confidence interval (CI), *P*-values and relative importance derived from the best-fit models. In the model, air temperature and precipitation indicate monthly mean air temperature and precipitation during mid-growing season (June to August), respectively, and plant biomass indicates the accumulated aboveground biomass before July.

**Supplementary References**

Beck, P.S.A., Atzberger, C., Høgda, K.A., Johansen, B. & Skidmore, A.K. (2006). Improved monitoring of vegetation dynamics at very high latitudes: a new method using MODIS NDVI. *Remote Sens. Environ.*, 100, 321–334.

Busetto, L., Colombo, R., Migliavacca, M., Cremonese, E., Meroni, M., Galvagno, M. *et al.* (2010). Remote sensing of larch phenological cycle and analysis of relationships with climate in the Alpine region. *Glob. Change Biol.*, 16, 2504–2517.

Pinzon, J.E. & Tucker, C.J. (2014). A non-stationary 1981-2012 AVHRR NDVI_3g_ time series. *Remote Sens.*, 6, 6929–6960.

Zhao, X., Hu, H., Shen, H., Zhou, D., Zhou, L., Myneni, R.B. *et al.* (2015). Satellite-indicated long-term vegetation changes and their drivers on the Mongolian Plateau. *Landscape Ecol.*, 30, 1599–1611.
